# Supplementary material for: Radial or Focal Extracorporeal Shock Wave Therapy in Lateral Elbow Tendinopathy: A Real-Life Retrospective Study
Source: Int J Environ Res Public Health. 2023 Feb 28;20(5):4371. doi: 10.3390/ijerph20054371 (PMC10002308; doi:10.3390/ijerph20054371)

Supplementary table S1: Baseline patients characteristics according to treatment-group. Data were reported as mean±SE and as absolute number and percentage, for continuous and categorical variables respectively.

	Radial ESWT 42	Focal ESWT 42	p-value
Age (years)	47.26±8.24	46.21±8.57	0.57
Female sex	25 (59.52)	21 (50.00)	0.38
Practicing Sport	9 (21.43)	13 (30.95)	0.32
Pain at right elbow	24 (57.14)	26 (61.90)	0.66
Smart working	20 (47.62)	13 (30.95)	0.12
Manual working	13 (30.95)	16 (38.10)	0.49
ESWT intensity (mj/mm^2)	-	0.12±0.01	
ESWT intensity (bar)	1.93±0.34	-	
Visual Analogic Scale-score	76.07±8.37	77.58±9.09	0.43
PICK muscle torque (Newton)	89.97±13.26	91.19±9.22	0.63
Mean muscle torque (Newton)	76.39±10.53	77.16±7.30	0.70
Patient-Rated Tennis Elbow Evaluation	62.87±4.46	62.89±4.13	0.98
Pain sub-scale (PRTEE)	28.50±2.45	29.33±2.91	0.15
Disability sub-scale (PRTEE)	34.37±2.74	33.54±2.12	0.13

Supplementary Table S2: Mixed Model: Variation of mean muscle strength during the follow-up according to treatment. The last follow-up (T12) was the reference for the comparison among time of the study.

			Model A<br>Unconditional Means Model	Model B<br>Unconditional Growth Model	Model C<br>Person level Model
Initial status	Intercept	γ_{00}	100.8±1.3 ***	112.8±1.7 ***	114.8±2.3 ***
	Treatment ES-R	γ_{01}			-3.9±3.3
Rate of change	Intercept (time)	γ_{10}		-6.2±0.2 ***	-6.5±0.3 ***
	Treatment*Time	γ_{11}			0.5±0.5
Level 1	Within person	δ^2_e	373.7±33.3***	42.4±4.7 ***	46.4±12.3 ***
Level 2	In initial status	δ^2_0	58.8±25.1**	45.9±12.2 ***	42.4±4.7 ***
	In rate of change	δ^2_1		1.1±0.3 ***	1.1±0.3 ***
	Covariance	δ_{01}		4.9±1.3 ***	4.9±1.3 ***
	Intraclass correlation		0.14	0.52	0.52
		ρ		0.33	0.35
		$R^2_{y,y1}$		0.53	0.53
		R^2_e		0.83	0.89
		R^2_0			0.01
		AIC	2981	2468	2462

Supplementary Figure S1: Correlation between VAS-score and Pain sub-scale in the Patient-Rated Tennis Elbow Evaluation (PRTEE). Linear regression analysis, R^2=0.63, p-value <0.001.

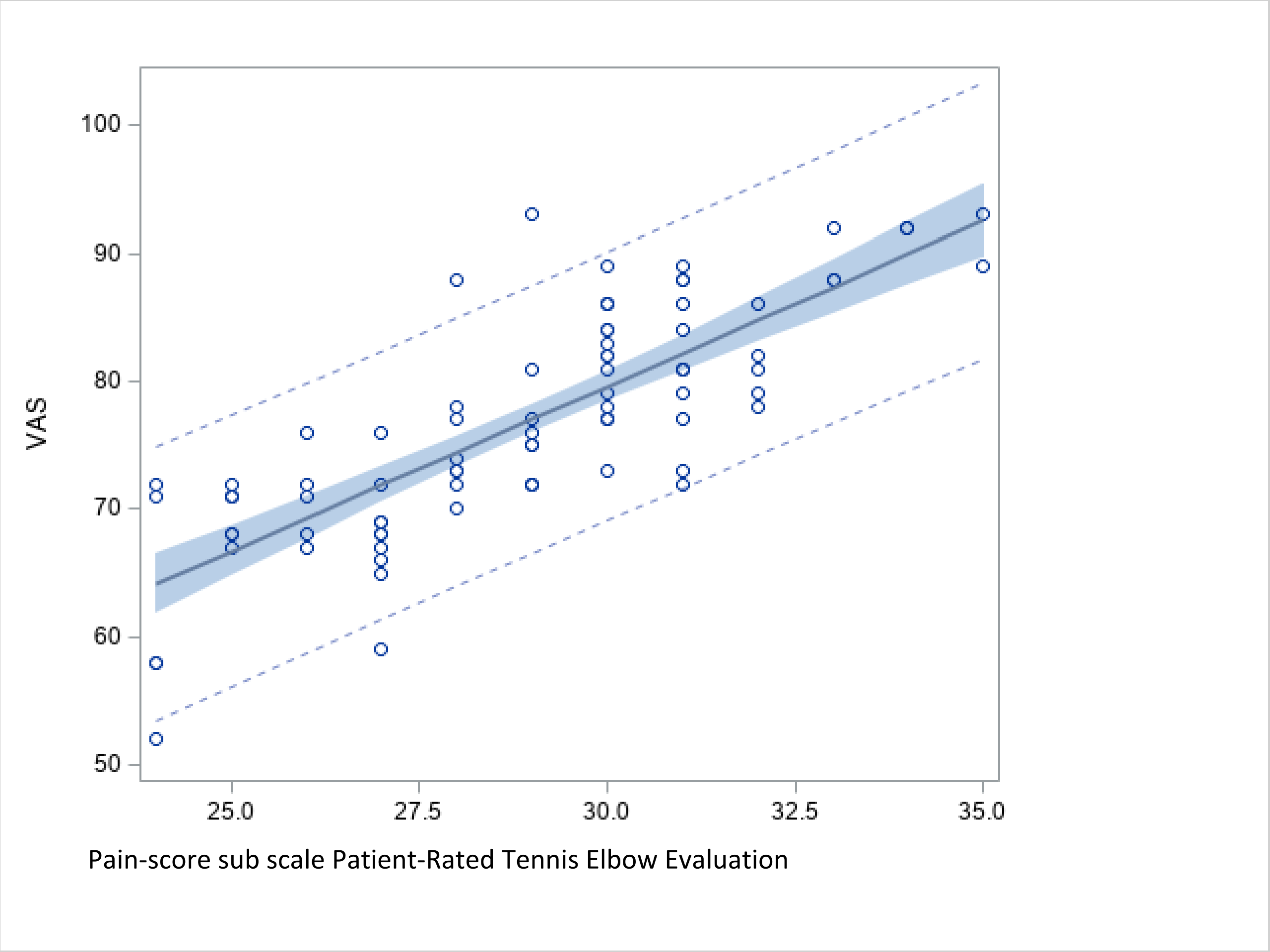

Supplement: Supplementary file 1 [file ijerph-20-04371-s001.zip › ijerph-2241318-supplementary.pdf]
